# Joint optimization for throughput fairness maximization in laser-powered UAV communications

**DOI:** 10.1371/journal.pone.0354743

**Published:** 2026-07-28

**Authors:** Guolong Zhang, Qian Dan, Yutian Zhang

**Affiliations:** Jiangxi University of Chinese Medicine, Nanchang, China; Southwest Jiaotong University, CHINA

## Abstract

This paper investigates a laser-powered UAV communication system. To maximize the minimum throughput among ground nodes, we jointly optimize communication scheduling, transmit power, and UAV trajectory. The resulting non-convex problem is decomposed into three subproblems and solved via successive convex approximation and iterative techniques. The simulation results show that our algorithm has fast and stable convergence.

## 1 Introduction

Unmanned aerial vehicles (UAVs) have the advantages of low cost, fast response time, flexibility, and convenience. Currently, UAVs are widely used in agriculture, transportation, disaster relief, and various public service domains due to their advantages [1,2]. They are also regarded as a key supporting tool for realizing the fifth-generation (5G) and above 5G wireless communication networks [3]. Consequently, UAVs-assisted wireless communication networks not only significantly enhance quality-of-service (QoS) of data transmission but also substantially reduces the operational and infrastructural costs associated with traditional communication systems. However, the limited carrying capacity of UAVs restricts their flight endurance. The energy supply of UAVs can increase their working time in the air. The laser charging technique is a better method for UAVs compared to conventional battery-powered solutions [4].

UAV-enabled communication systems have emerged as a prominent research frontier with diverse applications, including data collection [5], communication assistance [6,7], integrated sensing and communication (ISAC) [8,9], and near-field communication [10]. In Ref. [6], the UAV adopting the orthogonal frequency division multiple access technology acted as a relay to serve multiple user equipment (UEs). The total average end-to-end throughput was maximized by jointly optimizing the UAV trajectory and sub-channel allocation under the QoS requirements of UE pairs. Ref. [7] investigated the UAV-assisted downlink wireless network in an area with no-fly zones (NFZs). The aurhors maximize the sum rate by jointly optimizing power allocation and UAV’s flying trajectory while satisfying the power budget, user minimum rate, and the NFZs evasion constraints. Ref. [8] investigated a UAV-assisted ISAC system. The UAV trajectory is optimized to minimize the weighted sum of the predicted posterior Cramér-Rao bound. Ref. [9] also investigated UAV-enabled ISAC systems, a reinforcement learning algorithm was proposed to minimize the total transmit power by optimizing the resource allocation and UAV trajectory. In [10], the authors proposed a novel near-field beam tracking method for UAVs that utilizes an ISAC system. The achievable rate and tracking accuracy are enhanced via extended Kalman filter-based beam tracking and compensation for UAV jitter. In Ref. [11], the transmission scheduling and the trajectory for a wireless-powered UAV were jointly optimized to minimize the completion time in the data collection system. The broadcast nature of UAV line-of-sight transmission poses unique security challenges, prompting research into both covert and secure communications in non-orthogonal multiple access (NOMA)-based UAV-mobile edge computing (MEC) systems. In Ref. [12], a secure communication scheme was proposed for NOMA-based UAV-MEC systems against a flying eavesdropper, where the average security computation capacity was maximized subject to a minimum security requirement for each ground user. In Ref. [13], the authors proposed a covert communication scheme for NOMA-based UAV-MEC systems against an aerial warden, where the average computing capacity is maximized by jointly optimizing UAV trajectory and system resources. Battery constraints limit the energy that UAVs can carry, thereby restricting their flight endurance.

The high flight energy consumption of UAV-assisted communication has driven research into remote charging methods such as solar and, wireless charging. In Ref. [14], the UAV harvests the solar energy and charges the Internet of things (IoT) nodes with laser. The UAV three-dimensional (3D) trajectory and communication scheduling of IoT nodes were jointly optimized to maximize the residual energy subject to the transmission rate constraints. Ref. [15] investigated the physical layer security (PLS) of a wireless-powered UAV communication system. The PLS was enhanced by jointly optimizing the wireless charging duration, transmit power, and UAV trajectory under the battery capacity and energy-harvesting causal.

Laser-based wireless power transfer has a long transmission distance and high power density which has better anti-interference ability and stronger energy transmission efficiency. The free space optic (FSO) communication of the UAV is also studied in [16,17]. Ref. [16] investigated the multiple UAV trajectory optimal problem to extend the service time between the multiple ground terminals and UAVs with FSO communication link. Ref. [17] considered the aerial backhaul networks in which ground IoT nodes gather data and transmits to UAV or high-altitude platform stations with FSO link. The end-to-end outage probability was minimized by optimizing the trajectory of the UAV. Laser-powered UAVs have solved the defects of solar-powered UAVs that rely on light and the problem of wireless-powered UAVs with limited power and short distances. Ref. [18] explored the interdependence of UAV flight, energy consumption, and battery dynamics, and a low power and high gain scheme was proposed to modify the UAV operation. The laser-charging and accurate battery level were utilized to enhance flying time. The authors studied the laser charging station improve the flying time of UAV which acts as a mounted base station (BS), the power and bandwidth allocation were jointly optimized to maximize the UAV placement flight time and communications data rate in Ref. [19]. In Ref. [20], the authors proposed a laser charging drone-mounted base-station (DBS) framework. The DBS was charged by the macro base station (MBS). DBS and MBS both provided computing services to the ground UEs.

The performance analysis of laser-powered UAVs is also a hot research topic. In Ref. [21], stochastic geometry was utilized to model the complex communication system which consists of multiple terrestrial base stations, ground users, and UAVs charged by laser. The authors explored the energy-coverage trade-off in wireless networks, yielding a closed-form expression for the coverage probability. In [22], the authors considered the laser charging UAV-aided communication network with wireless information and power transfer system. The performance in terms of user outage probability, throughput, and average harvested power were analyzed and their expression was derived. A performance analysis was conducted in Ref. [23], where a laser-powered UAV acting as a mounted BS to provide communication with non-orthogonal multiple access for multiple IoT nodes. The closed-form expressions of the outage probabilities and others were derived and verified for accuracy. Literature [24] investigated the performance analysis of a dual-hop decode-and-forward (DF) relay system, in which a UAV acts as a laser-charged relay. The maximum achievable rate or ergodic capacity, the outage probability, and the error probability are analyzed and derived the closed-form expression.

Laser-powered UAV-assisted relay systems can effectively improve system performance. In Ref. [25], the UAV was charged by a laser station and acted as a DF relay. The total decoding error rate was minimized by jointly optimizing resource allocation under the constraints of power control, trajectory planning, and energy harvesting constraints, considering geometric loss in the laser model. In Ref. [26], the authors considered the laser charging UAV relay system hovering-only UAV mobility and a time-averaged laser model, the EE of UAV was maximized by jointly optimizing the transmit powers and UAV trajectory under the energy-causality and sum rate constraints. The authors investigated the laser charging UAV-assist relay IoT haeterogeneous network system, the number of the uplink transmission IoT nodes was maximized under the QoS of the statistics and outage probability requirements of the IoT devices and energy causality constraints in Ref. [27]. In Ref. [28], the UAV trajectory and laser charging power were jointly optimized to minimize the power consumption of the charging station while taking the QoS of data transmission into account in relay systems.

Laser-powered UAV-assisted data collection has attracted considerable scholarly attention. Ref. [29] studied multiple UAV-assisted wireless sensor networks (WSN) charged by multiple mobile unmanned vehicles (MUVs) loaded with laser transmitters, where each UAV not only communicates with the WSN but also transfers energy to it. The travel plan of UAVs was optimized to charge WSNs which ensure the remaining energy and minimize the maximum time consumption. Ref. [30] proposed a modified multi-objective reinforcement learning algorithm, the authors maximized energy efficiency (EE) and the number of tasks collected by optimizing the UAV’s flight trajectories. In [31], a laser-powered UAV-assisted IoT collection data systems was studied with two-phase alternating trajectory optimization, the UAV trajectory and resource allocation were jointly optimized to maximize the EE of the system. The authors in [32] investigated the large-scale wireless rechargeable sensor networks that were charged and data collected by a laser-powered UAV, using hovering and sequential waypoints (straight lines) to maximize the number of IoT devices by optimizing 3D hovering points. Ref. [33] and [34] considered a UAV-aided data collection system that was charged by ground laser station and the IoT, the authors minimized the energy consumption of laser source by optimizing UAV trajectory under the constraint of minimum collected data. Ref. [35] investigated laser-powered UAV-assisted IoT networks, the authors jointly optimized UAV trajectories and laser charging strategies to minimize peak age of information(AoI). Ref. [36] investigates a hybrid laser-battery-powered UAV data collection system, optimizing the UAV’s 3D trajectory, radiated power, and temporal battery usage profile to enhance system performance.

The main contributions of this paper are summarized as follows:

1) We investigated a scenario in which a laser-powered UAV transmits information to ground nodes (GNs), maximizing the minimum GN throughput under the premise of GN fairness. Different from existing battery-powered UAV works (e.g., [5–13]), the continuity of laser powering eliminates the need for the UAV to return for charging, which fundamentally changes the nature of the energy constraint and enables long-duration continuous service.
2) Compared with wireless-powered UAV works (e.g., [14,15]), this study introduces a laser charging constraint mechanism, enhancing system stability by establishing multi-dimensional constraint conditions. Different from the hovering‑only or sequential‑waypoint designs adopted in [27,29,32], our work employs continuous trajectory optimization. Unlike the works in [25,28,30,33,34,36] that also consider geometric attenuation, our laser energy harvesting model additionally accounts for multi‑laser‑station switching. Moreover, compared with all the above references, we attempt to incorporate user fairness (max‑min throughput fairness) into the optimization framework.
3) For the non-convex problem of parameter coupling in the original problem, we adopt the block coordinate descent (BCD) scheme to decouple the original problem and decompose it into three solvable subproblems. Each subproblem is transformed into a convex problem by utilizing the successive convex approximation (SCA) method, and an iterative solution is adopted. The proposed iterative algorithm has good convergence and stability. The uniqueness of this solution framework lies in the fact that due to the tight coupling between laser energy constraints and communication constraints via the UAV’s position, standard optimization methods for laser-powered UAVs cannot be directly applied. Our BCD-SCA decomposition strategy is tailored to this coupling structure.

To better illustrate the technical differences between our work and existing laser‑powered UAV studies, particularly regarding trajectory design and laser energy harvesting models, we provide a quantitative comparison in Table 1. It is worth noting that our work differs fundamentally from [27] and [32] in two key aspects. First, regarding trajectory design: [27] assumes a hovering UAV (no trajectory optimization), and [32] adopts sequential hovering at cluster centers, whereas our work jointly optimizes the continuous 3D trajectory (position, velocity, and acceleration). Second, regarding the laser energy harvesting model: [27] uses a time-averaged model (valid only for hovering), and [32] employs a simplified fixed-efficiency model with only exponential attenuation; in contrast, our model is instantaneous, distance-dependent, and includes beam divergence, geometric loss, atmospheric attenuation, and dynamic selection of the nearest laser station. Moreover, unlike [27] and [32], our work explicitly targets max-min throughput fairness among GNs.

**Table 1:** Comparison of related works.

Reference	UAV mobility design	Laser model – Geometric loss	Laser model – Multi‑station switching	Laser model – Time‑averaged	User fairness	Optimization objectives	Main parameters
[25]	Continuous trajectory optimization	✓				minimize the total decoding error rate	Resource allocation,power control, trajectory
[26]	Continuous trajectory optimization					maximize the weighted sum of the energy efficiency	Transmit powers trajectory
[27]	Hovering only			✓		maximize the number of the connected IoT devices with the UAV relay for uplink data transmission	Device connectivity selection, UAV transmit power, time-slot allocation among devices, and laser charging duration
[28]	Continuous trajectory optimization	✓				minimize the power consumption of the charging station	Trajectory and charging power
[29]	Hovering, sequential waypoints (straight lines)					minimize the maximum time for UAVs to charge the WSN.	Optimal travel plan of UAVs and MUVs
[30]	Continuous trajectory optimization	✓				maximize both the energy efficiency of the UAV and the number of tasks collected	Trajectory, communication resources allocation
[31]	Two‑phase alternating trajectory optimization					maximize system energy efficiency	Wireless communication resources, trajectory
[32]	Hovering,sequential waypoints (straight lines)					maximize the number of energy-sufficient SNs that transmit back data	3D hovering points,visiting order
[33]	Continuous trajectory optimization	✓				maximize average collection data, minimize the laser energy expenditure	Radiated laser power, radiated power, trajectory
[34]	Continuous trajectory optimization	✓				maximize average collection data	Radiated laser power, radiated power, trajectory
[35]	Continuous trajectory optimization					minimize the peak AoI	Trajectory, laser charging strategies
[36]	Continuous trajectory optimization	✓				maximize destination battery level and minimize the laser energy consumption	UAV trajectory, the radiated power profiles of both the laser source and UAV, the battery usage profile
Our Work	Continuous trajectory optimization	✓	✓		✓	maximize the minimum throughput	Communication scheduling, Transmit power, trajectory

The remaining organization of this paper is summarized as follows. Sect. 2 introduces the system model of the laser-power UAV-assisted information transmission systems considering the fairness of GNs. In Sect. 3, an efficient iterative algorithm that integrates BCD with SCA is proposed to solve the formulated problem. In Sect. 4, the simulation results of the algorithm are analyzed. Finally, Sect. 5 summarizes the work of the paper.

## 2 System model

**Fig 1:**
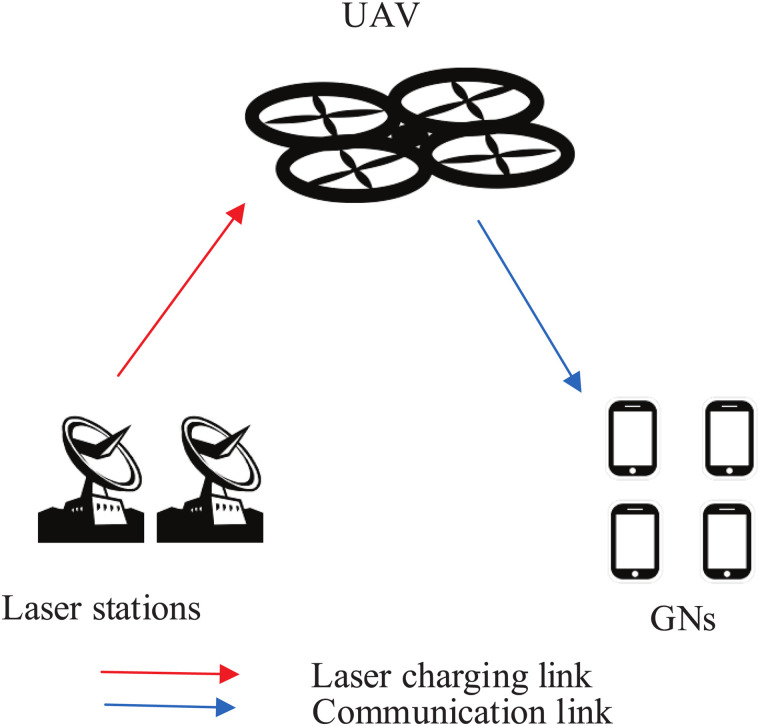
System model consisting of *K* GNs, *M* laser stations and a UAV.

We considered a laser-powered UAV-assisted communication system consisting of *M* laser stations and *K* GNs in Fig 1. The UAV is assumed to be equipped with dual antennas: one for information transmission and the other for laser energy harvesting, and the two antennas can work simultaneously. Both the laser station and the GNs are single-antenna. To facilitate the solution, we divide the total flight time *T* into *N* equal parts, and the time slot of each is δ. In each slot, the UAV is powered by one laser station and serves one GN.

It is assumed that the UAV flies with a constant altitude *H*, and the 3D position in slot *n* is (x[n],y[n],H), its two-dimensional (2D) coordinates is denoted as q[n]=(x[n],y[n]),. The laser stations and GNs are on the ground, and the 2D coordinates of *m*-th laser and *k*-th node are respectively denoted by $q_{m}$ and $q_{k}$. For ease of reference, the main symbol notations are summarized in Table 2. Assuming the UAV–GN link is line-of-sight (LoS) [5], and there is no obstacle between laser stations and the UAV [24,30].

**Table 2:** List of symbol notations.

Notation	Description
${𝐪}_{I}$	Initial horizontal location of UAV
${𝐪}_{F}$	Final horizontal location of UAV
𝐪[n]	Horizontal location of UAV
${𝐪}_{m}$	The location of *m*-th laser station
${𝐪}_{k}$	The location of *k*-th GN
$\sigma^{2}$	The noise power
$\beta_{0}$	The channel power gain at the reference distance
*P*	The average communication power
$P_{s}$	The transmit power
$\lambda_{k}$	Communication scheduling parameter
$\alpha_{m}$	Energy supply scheduling parameter
ε	Algorithm convergence precision
*P*_0_	Blade profile power
$P_{i}$	Induced power
*v*_0_	The mean rotor induced velocity
$U_{tip}$	Tip speed of the rotor blade
*d*_0_	Fuselage drag ratio
ρ	Air density
*s*	Rotor solidity
*A*_0_	Rotor disc area
$v_{\max}$	Maximum horizontal speed
$a_{\max}$	Maximum horizontal acceleration
*A*	The area of collection lens
*D*	Initial laser beam’s size
Δθ	The angular spread
φ	The optical efficiency of transmission and reception
χ	The combined transmission receiver’s optical efficiency
α	The medium’s attenuation coefficient
*w*	Laser energy harvesting efficiency

The distance between the UAV and *m*-th laser station is


$$\begin{array}{c} d_{m}[n]=\sqrt{{\left\|q[n]-q_{m}\right\|}^{2}+H^{2}} \end{array}$$ (1)

The distance between the UAV and *k*-th GN is expressed as


$$\begin{array}{c} d_{k}[n]=\sqrt{{\left\|q[n]-q_{k}\right\|}^{2}+H^{2}} \end{array}$$ (2)

The channel gain between the UAV and *k*-th GN is modeled as


$$\begin{array}{c} h_{k}^{2}[n]=\frac{\beta_{0}}{{\left\|q[n]-q_{k}\right\|}^{2}+H^{2}} \end{array}$$ (3)

where $\beta_{0}$ denotes the reference channel power gain at 1-meter distance.

The achievable rate for GN *k* in slot *n* is


$$\begin{array}{c} R_{k}[n]=\lambda_{k}[n]\log_{2}\left(1+\frac{P_{s}[n]h_{k}^{2}[n]}{\sigma^{2}}\right) \end{array}$$ (4)

where $\lambda_{k}[n]$ is binary number which $\lambda_{k}[n]=1$ denote the UAV communicating with *k*-th GN in *n* slot and $\lambda_{k}[n]=0$ denote the UAV is not communicating with *k*-th GN in *n* slot, $P_{s}$ is the communication transmission power of the UAV, $\sigma^{2}$ is Gaussian white noise power.

The energy consumed during UAV flight is substantial, and the energy consumption model for rotor UAVs is formulated as [20]


$$\begin{array}{c} P_{F}\left(\left\|v\left[n\right]\right\|\right)=P_{0}\left(1+\frac{3{\left\|v\left[n\right]\right\|}^{2}}{U_{tip}^{2}}\right)+P_{i}{\left(\sqrt{1+\frac{{\left\|v\left[n\right]\right\|}^{4}}{4v_{0}^{4}}}-\frac{{\left\|v\left[n\right]\right\|}^{2}}{2v_{0}^{2}}\right)}^{\frac{1}{2}}+\frac{1}{2}d_{0}\rho sA_{0}{\left\|v\left[n\right]\right\|}^{3} \end{array}$$ (5)

where *P*_0_ and $P_{i}$ represent the blade profile power and induced power of the rotor UAV in hover state, $U_{tip}$ represents the tip speed of the rotor blade, *v*_0_ represents the average rotor-induced speed of the UAV in a hover state, v[n] is the flight speed of the UAV in *n*-th time slots. In addition, *d*_0_ and ρ represent the fuselage damping ratio and air density respectively, *s* is the solidity of the rotor, and *A*_0_ represents the turntable area.

The flight energy consumption of the UAV is powered by the laser station during the flight, and the intensity of the laser energy power received by the UAV is expressed as [25]


$$\begin{array}{c} S[n]=\varphi \frac{A}{{\left(D+d_{m}[n]\Delta \theta \right)}^{2}}\chi e^{-\alpha d_{m}[n]} \end{array}$$ (6)

where φ is the optical efficiency of transmission and reception, *A* is the area of the receiving telescope or acquisition lens, *D* is the size of the initial laser beam, Δθ is the transmission angle, χ represents the overall optical efficiency of the entire signal chain from transmitter to receiver, α is the attenuation coefficient of the medium. Assuming that the efficiency of laser energy power acquisition is w∈[0,1], the laser-charged power of the UAV is expressed as


$$\begin{array}{c} P_{h}[n]=wS[n]=\frac{C\varphi e^{-\alpha d_{m}[n]}}{{\left(D+d_{m}[n]\Delta \theta \right)}^{2}} \end{array}$$ (7)

where C=wAχ. It is proposed that the UAV be charged by the laser station that is closest to the UAV in each time slot. For tractability, we assume that the UAV can continuously receive laser power from that nearest station without blockage or alignment errors. In practice, laser power transfer is sensitive to pointing accuracy, atmospheric attenuation, and LoS obstructions. We acknowledge that under adverse conditions (e.g., LoS blockage or misalignment), this assumption may not hold. However, it is justified as follows. As the first study, to the best of our knowledge, that unifies laser charging and max-min fairness for UAV communications, our primary goal is to establish a tractable analytical framework while retaining the key characteristic of laser charging that distance dependent power loss. Therefore, the current model serves as an ideal-case performance benchmark under favorable LoS conditions. When the link to the nearest laser station is unavailable, a natural extension is to introduce a dynamic laser station scheduling mechanism that switches the UAV to alternative available stations. This is identified as an important direction for future research.

The distance between the UAV and the energy supply laser station is utilized to denote the energy supply constraint, the constraint relationship between UAV and *m*-th laser transmitter is expressed as


$$\begin{array}{c} \alpha_{m}[n]=\{\begin{array}{cc} 1, & m=\text{arg}\underset{j\in M}{\min}d_{j}[n]\\ 0, & otherwise \end{array} \end{array}$$ (8)

The laser energy harvested by the UAV is expressed as


$$\begin{array}{c} P_{L}[n]=\sum_{m=1}^{M}\alpha_{m}[n]\frac{C\varphi e^{-\alpha d_{m}[n]}}{{\left(D+d_{m}[n]\Delta \theta \right)}^{2}} \end{array}$$ (9)

## 3 Problem formulation

The UAV relies on the energy harvested from the laser station to power its flight, and the UAV transmits information between nodes on the ground during flight. We pay more attention to the fairness among GNs, and the optimization problem is expressed as


$$\begin{array}{cc} \left(P1\right)\underset{\lambda_{k}[n],P_{s}\left[n\right],q[n]}{\max} & \underset{k}{\min}\sum_{n=1}^{N}R_{k}[n] \end{array}$$ (10a)

$$\begin{array}{cc} s.t. & \sum_{k=1}^{K}\lambda_{k}[n]=1 \end{array}$$ (10b)

$$\begin{array}{cc} & \lambda_{k}[n]\in \left\{0,1\right\} \end{array}$$ (10c)

$$\begin{array}{cc} & \frac{1}{N}\sum_{n=1}^{N}P_{s}\left[n\right]\leq P \end{array}$$ (10d)

$$\begin{array}{cc} & \sum_{l=1}^{n}P_{F}[l]\leq \sum_{l=1}^{n}P_{L}[l],1\leq n\leq N \end{array}$$ (10e)

$$\begin{array}{cc} & q[0]=q_{I} \end{array}$$ (10f)

$$\begin{array}{cc} & q[N]=q_{F} \end{array}$$ (10g)

$$\begin{array}{cc} & q[n+1]=q[n]+v[n]\delta_{t}+\frac{1}{2}a[n]\delta_{t}^{2} \end{array}$$ (10h)

$$\begin{array}{cc} & v[n+1]=v[n]+a[n]\delta_{t} \end{array}$$ (10i)

$$\begin{array}{cc} & v[1]=v[N] \end{array}$$ (10j)

$$\begin{array}{cc} & \left|v[n]\right|\leq v_{\max} \end{array}$$ (10k)

$$\begin{array}{cc} & \left|a[n]\right|\leq a_{\max} \end{array}$$ (10l)

The objective function of the optimization problem is to maximize the minimum the GNs’ total transmission rate during the UAV flight. Eqs. (10b) and (10c) are the communication constraints between the UAV and the GN at *n* slot, (10b) denotes that UAV only communicates with a node at *n* slot and (10c) is the parameter threshold limit. (10d) is the average communication power constraints of the UAV, (10e) indicates that the harvested energy is greater than the flight energy, so as to ensure the supply of flight energy. (10f) and (10g) are the initial and final point constraints. (10h) and (10i) is the relationship between the UAV’s trajectory, velocity, and acceleration. (10j) is the relationship between the initial and final velocity. (10k) and (10l) are velocity and acceleration constraints, $v_{\max}$ and $a_{\max}$ are the maximum velocity and acceleration, respectively. The above problem cannot be solved directly due to the coupling of variables and the optimization objective, which constrain the non-convexity of (10a), (10c), and (10e). Therefore, the original problem is decoupled into three sub-problems, the communication scheduling factor optimization sub-problem, the communication transmitting power sub-problem, and the UAV flight trajectory optimization sub-problem, and then the sub-optimal solution is obtained by iterating respectively.

### 3.1 Communication scheduling optimization

Communication scheduling is still a 0–1 integer programming problem, and it is difficult to solve it directly. The scheduling factor can be relaxed to continuous variable constraints. With the given $P_{s}\left[n\right]$ and q[n], the communication scheduling optimization sub-problem is expressed as


$$\begin{array}{cc} \left(P1.1a\right) & \underset{\lambda_{k}[n]}{\max}\underset{k}{\min}\sum_{n=1}^{N}\lambda_{k}[n]R_{k}\left[n\right]\\ & \left(10b),(10c\right) \end{array}$$ (11a)

The optimization objective of the above problem cannot be solved directly, we introduce the relaxation variable *t*_1_, relax $\lambda_{k}[n]$ to be continuous, and the problem is rewritten as


$$\begin{array}{cc} \left(P1.1b\right) & \underset{\lambda_{k}[n],t_{1}}{\max}t_{1} \end{array}$$ (12a)

$$\begin{array}{cc} s.t. & 0\leq \lambda_{k}[n]\leq 1 \end{array}$$ (12b)

$$\begin{array}{cc} & \sum_{n=1}^{N}\lambda_{k}[n]R_{k}\left[n\right]\geq t_{1}\\ & (10b), \end{array}$$ (12c)

The constraints of the above problems are all linear expressions of variables, and the objective function is also a first-order function of variables. Therefore, (P1.1b) is a linear programming problem, which can be easily solved via standard linear programming methods. The communication scheduling factor can be obtained by solving it.

### 3.2 Transmit power optimization

With the given $\lambda_{k}[n]$ and q[n], the transmit power optimization sub-problem $P_{s}\left[n\right]$ is expressed as


$$\begin{array}{cc} \left(P1.2a\right) & \underset{P_{s}\left[n\right]}{\max}\underset{k}{\min}\sum_{n=1}^{N}\lambda_{k}\left[n\right]\log_{2}\left(1+\frac{P_{s}\left[n\right]\gamma_{0}}{d_{k}^{2}[n]}\right)\\ s.t. & (10d), \end{array}$$ (13a)

where $\gamma_{0}=\frac{\beta_{0}}{\sigma^{2}}$. The optimization objective of the above problem cannot be solved directly. To solve it, the relaxation variable *t*_2_ is introduced and the problem is rewri*tt*en as


$$\begin{array}{cc} \left(P1.2b\right) & \underset{P_{s}[n],t_{2}}{\max}t_{2} \end{array}$$ (14a)

$$\begin{array}{cc} s.t. & \sum_{n=1}^{N}\lambda_{k}\left[n\right]\log_{2}\left(1+\frac{P_{s}\left[n\right]\gamma_{0}}{d_{k}^{2}[n]}\right)\geq t_{2}\\ & (10d), \end{array}$$ (14b)

Since the left-hand side (LHS) of (14b) is concave in $P_{s}$, and the other constraints also meet the convex constraint. Therefore, subproblem 2 is a standard convex problem, which can be readily solved using the CVX toolbox.

### 3.3 Trajectory optimization

With the given $\lambda_{k}[n]$ and $P_{s}\left[n\right]$, the trajectory optimization sub-problem q[n] is expressed as


$$\begin{array}{cc} \left(P1.3a\right) & \underset{q[n]}{\max}\underset{k}{\min}\sum_{n=1}^{N}\lambda_{k}[n]\log_{2}\left(1+\frac{P_{s}\left[n\right]\gamma_{0}}{{\left\|q[n]-q_{k}\right\|}^{2}+H^{2}}\right) \end{array}$$ (15a)

$$\begin{array}{cc} s.t. & \sum_{l=1}^{n}\left(P_{0}\left(1+\frac{3{\left\|v\left[l\right]\right\|}^{2}}{U_{tip}^{2}}\right)+P_{i}{\left(\sqrt{1+\frac{{\left\|v\left[l\right]\right\|}^{4}}{4v_{0}^{4}}}-\frac{{\left\|v\left[l\right]\right\|}^{2}}{2v_{0}^{2}}\right)}^{\frac{1}{2}}+\frac{1}{2}d_{0}\rho sA_{0}{\left\|v\left[l\right]\right\|}^{3}\right)\\ & \leq \sum_{l=1}^{n}\sum_{m=1}^{M}\alpha_{m}[l]\frac{C\varphi e^{-\alpha \left({\left\|q[l]-q_{m}\right\|}^{2}+H^{2}\right)}}{{\left(D+\left({\left\|q[l]-q_{m}\right\|}^{2}+H^{2}\right)\Delta \theta \right)}^{2}}\\ & \left(10f)-(10l\right) \end{array}$$ (15b)

Due to the non-convexity of the optimization objective and constraints (15b), this subproblem is not a standard convex problem, and solving this problem is full of challenges. To facilitate solving this problem, a slack variable *t*_3_ is introduced, and this subproblem can be expressed as


$$\begin{array}{cc} \left(P1.3b\right) & \underset{q[n],v[n],a[n],t_{3}}{\max}t_{3} \end{array}$$ (16a)

$$\begin{array}{cc} s.t. & \sum_{n=1}^{N}\lambda_{k}[n]\log_{2}\left(1+\frac{P_{s}\left[n\right]\gamma_{0}}{{\left\|q[n]-q_{k}\right\|}^{2}+H^{2}}\right)\geq t_{3} \end{array}$$ (16b)

$$\begin{array}{cc} & \sum_{l=1}^{n}\left(P_{0}\left(1+\frac{3{\left\|v\left[l\right]\right\|}^{2}}{U_{tip}^{2}}\right)+P_{i}{\left(\sqrt{1+\frac{{\left\|v\left[l\right]\right\|}^{4}}{4v_{0}^{4}}}-\frac{{\left\|v\left[l\right]\right\|}^{2}}{2v_{0}^{2}}\right)}^{\frac{1}{2}}+\frac{1}{2}d_{0}\rho sA_{0}{\left\|v\left[l\right]\right\|}^{3}\right)\\ & \leq \sum_{l=1}^{n}\sum_{m=1}^{M}\alpha_{m}[l]\frac{C\varphi e^{-\alpha \left({\left\|q[l]-q_{m}\right\|}^{2}+H^{2}\right)}}{{\left(D+\left({\left\|q[l]-q_{m}\right\|}^{2}+H^{2}\right)\Delta \theta \right)}^{2}}\\ & \left(10f)-(10l\right) \end{array}$$ (16c)

In the above problem, (16b) is a non-convex constraint on q[n], which is troublesome to deal with, but if we treat ${\left\|q[n]-q_{k}\right\|}^{2}+H^{2}$ as a whole, then the constraint is a convex function on ${\left\|q[n]-q_{k}\right\|}^{2}+H^{2}$. To make it easier to solve, we introduce a relaxation variable $\left\{U_{k}|U_{k}\geq {\left\|q[n]-q_{k}\right\|}^{2}+H^{2}\right\}$. In this case, the LHS of (16b) does not meet the convex constraint, the first-order Taylor expansion is utilized to obtain the lower bound, which is expressed as


$$\begin{array}{cc} \log_{2}\left(1+\frac{P_{s}\left[n\right]\gamma_{0}}{U_{k}\left[n\right]}\right)\geq & \frac{-P_{s}\left[n\right]\gamma_{0}}{\ln2\left(U_{k}^{\left(j\right)}\left[n\right]+P_{s}\left[n\right]\gamma_{0}\right)\left(U_{k}^{\left(j\right)}\left[n\right]\right)}\left(U_{k}\left[n\right]-U_{k}^{\left(j\right)}\left[n\right]\right)+\\ & \;\log_{2}\left(1+\frac{P_{s}\left[n\right]\gamma_{0}}{U_{k}^{\left(j\right)}\left[n\right]}\right)=\varphi_{k}^{lb}\left[n\right] \end{array}$$ (17)

The constraint (16c) is also a non-convex constraint, the LHS and the right-hand side (RHS) of the constraint are non-convex. The LHS of Eq. (16c) is a non-convex function on velocity, in order to solve this problem, we introduce relaxation variables $v_{l}$, which is expressed as


$$\begin{array}{c} v_{l}^{2}\geq {\left\|v\left[l\right]\right\|}^{2} \end{array}$$ (18)

The power consumption of the UAV flight is rewritten as


$$\begin{array}{c} P\left(v_{l}\right)=P_{0}\left(1+\frac{3v_{l}^{2}}{U_{tip}^{2}}\right)+P_{i}{\left(\sqrt{1+\frac{v_{l}^{4}}{4v_{0}^{4}}}-\frac{v_{l}^{2}}{2v_{0}^{2}}\right)}^{\frac{1}{2}}+\frac{1}{2}d_{0}\rho sA_{0}v_{l}^{3} \end{array}$$ (19)

The second term in the formula is still non-convex, in order to facilitate the solution, we introduce the relaxation variable $u_{l}$, and the second term in the above formula is rewritten as


$$\begin{array}{c} u_{l}^{2}\geq \sqrt{1+\frac{v_{l}^{4}}{4v_{0}^{4}}}-\frac{v_{l}^{2}}{2v_{0}^{2}} \end{array}$$ (20)

To make it easier to solve, (20) is equivalent to


$$\begin{array}{c} \frac{1}{u_{l}^{2}}\leq u_{l}^{2}+\frac{v_{l}^{2}}{v_{0}^{2}} \end{array}$$ (21)

The Eq. (21) remains non-convex and can be solved by SCA schemes. The RHS of the Eq. (21) is respectively convex function about $u_{l}$ and $v_{l}$, the lower bound of the RHS of the Eq. (21) by Taylor expansion


$$\begin{array}{c} \begin{array}{c} u_{l}^{2}+\frac{v_{l}^{2}}{v_{0}^{2}}\geq {\left(u_{l}^{\left(j\right)}\right)}^{2}+2u_{l}^{\left(j\right)}\left(u_{l}-u_{l}^{\left(j\right)}\right)+\frac{1}{v_{0}^{2}}{\left(v_{l}^{\left(j\right)}\right)}^{2}+2\frac{v_{l}^{\left(j\right)}}{v_{0}^{2}}\left(v_{l}-v_{l}^{\left(j\right)}\right)=\phi_{u}^{lb}[l] \end{array} \end{array}$$ (22)

The concavity of the RHS of (16c) about q[l] is not determined, if $\left({\left\|q[l]-q_{m}\right\|}^{2}+H^{2}\right)$ acts as a whole, the RHS of (16c) is a convex function about $\left({\left\|q[l]-q_{m}\right\|}^{2}+H^{2}\right)$, and the relaxation variable $\left\{U_{m}|U_{m}[l]\geq {\left\|q[l]-q_{m}\right\|}^{2}+H^{2}\right\}$ is introduced, the harvest energy is


$$\begin{array}{c} P_{L}[l]=\sum_{l=1}^{n}\alpha_{m}[l]\frac{C\varphi e^{-\alpha \left(U_{m}[l]\right)}}{{\left(D+U_{m}[l]\Delta \theta \right)}^{2}} \end{array}$$ (23)

Eq. (23) is about convex functions with $U_{m}$, the lower bounds can be obtained by utilizing the first-order Taylor approximation,


$$\begin{array}{cc} & \frac{C\varphi e^{-\alpha \sqrt{U_{m}\left[l\right]}}}{{\left(D+\sqrt{U_{m}\left[l\right]}\Delta \theta \right)}^{2}}\geq \frac{C\varphi e^{-\alpha \sqrt{U_{m}^{\left(j\right)}\left[l\right]}}}{{\left(D+\sqrt{U_{m}^{\left(j\right)}\left[l\right]}\Delta \theta \right)}^{2}}\\ & -\left(\frac{\alpha C\varphi e^{-\alpha \left(\sqrt{U_{m}^{\left(j\right)}\left[l\right]}\right)}}{2\sqrt{U_{m}^{\left(j\right)}\left[l\right]}{\left(D+\sqrt{U_{m}^{\left(j\right)}\left[l\right]}\Delta \theta \right)}^{2}}+\frac{\Delta \theta C\varphi e^{-\alpha \sqrt{U_{m}^{\left(j\right)}\left[l\right]}}}{\sqrt{U_{m}^{\left(j\right)}\left[l\right]}{\left(D+\sqrt{U_{m}^{\left(j\right)}\left[l\right]}\Delta \theta \right)}^{3}}\right)\left(U_{m}\left[l\right]-U_{m}^{\left(j\right)}\left[l\right]\right)\\ & =\phi_{PL}^{lb}\left[l\right] \end{array}$$ (24)

In summary, the optimization problem can be translated into


$$\begin{array}{cc} \left(P1.3c\right) & \underset{q[n],v[n],a[n],U_{m},U_{k},u_{l},v_{l},t_{3}}{\max}t_{3} \end{array}$$ (25a)

$$\begin{array}{cc} s.t. & \sum_{n=1}^{N}\lambda_{k}[n]\varphi_{k}^{lb}[n]\geq t_{3} \end{array}$$ (25b)

$$\begin{array}{cc} & \sum_{l=1}^{n}\left(P_{0}\left(1+\frac{3v_{l}^{2}}{U_{tip}^{2}}\right)+P_{i}u_{l}+\frac{1}{2}d_{0}\rho sAv_{l}^{3}\right)\leq \sum_{l=1}^{n}\phi_{PL}^{lb}[l] \end{array}$$ (25c)

$$\begin{array}{cc} & \frac{1}{u_{l}^{2}}\leq \phi_{u}^{lb}[l] \end{array}$$ (25d)

$$\begin{array}{cc} & U_{m}\left[l\right]\geq {\left\|q[l]-q_{m}\right\|}^{2}+H^{2} \end{array}$$ (25e)

$$\begin{array}{cc} & U_{k}\left[n\right]\geq {\left\|q[n]-q_{k}\right\|}^{2}+H^{2}\\ & \left(10f)-(10l\right) \end{array}$$ (25f)

This problem is a standard convex problem that can be solved using the CVX toolbox.

### 3.4 Overall algorithm

The original problem was decomposed into three interdependent subproblems, and SCA and iteration techniques were utilized to solve the above three subproblems. The convergence threshold is set as ε, and the above-proposed algorithm is summarized as Algorithm 1.

**Algorithm 1 An iterative algorithm for solving (P1)**

1: **Input:** $q_{k}$, $q_{m}$, $q_{I}$, $q_{F}$, $a_{\max}$, $v_{\max}$, *H*

2: **Output:** $\lambda_{k}\left[n\right]$, $P_{s}[n]$, q[n]

3: Initialization: set initial variables; tolerance ε>0 and iteration number *j* = 0

4: **Repeat**

5: With given $q^{\left(j\right)}[n]$, $P_{s}^{\left(j\right)}[n]$, obtain $\lambda_{k}^{\left(j+1\right)}[n]$ by solving problem (P1.1b)

6: With given $q^{\left(j\right)}[n]$, $\lambda_{k}^{\left(j+1\right)}[n]$, update $P_{s}^{\left(j+1\right)}[n]$ by solving problem (P1.2b)

7: With given $\lambda_{k}^{\left(j+1\right)}[n]$, $P_{s}^{\left(j+1\right)}[n]$, update $q^{\left(j+1\right)}[n]$ by solving problem (P1.3c) and obtain suboptimal value

8: *j* = *j* + 1

9: **Until** the algorithm convergence

10: Obtain the optimal value

The SCA method can only guarantee convergence to a local optimum due to problem non-convexity. However, this is practically sufficient because: 1) global optimality would require exponential-time algorithms, which are infeasible; 2) our method significantly outperforms heuristic baselines; 3) multi-start yields consistent objective values, suggesting near-global optimality. Therefore, the proposed method balances optimality and efficiency.

The initial trajectory of the UAV is set in a straight line from $q_{I}$ to $q_{F}$. Within a given time *T* and we divide *T* into *N* time slots, the initial trajectory of the UAV is expressed as


$$\begin{array}{c} q[n]=q_{I}+\frac{n}{N}(q_{F}-q_{I}),n=0,1,2,...,N \end{array}$$ (26)

**Convergence Analysis**: The convergence of **Algorithm 1** is proved as follows. Define $R\left(\lambda_{k}\left[n\right],P_{s}\left[n\right],q\left[n\right]\right)$ as the objective function of ${𝒫}_{1}$, and *j* denotes the iteration index.

With the given $P_{s}^{\left(j\right)}\left[n\right]$, $q^{\left(j\right)}\left[n\right]$, solving the communication scheduling optimization subproblem yields $\lambda_{k}^{\left(j\right)}\left[n\right]$ that improves the maximum minimum throughput:


$$\begin{array}{c} R\left(\lambda_{k}^{\left(j\right)}\left[n\right],P_{s}^{\left(j\right)}\left[n\right],q^{\left(j\right)}\left[n\right]\right)\leq R\left(\lambda_{k}^{\left(j+1\right)}\left[n\right],P_{s}^{\left(j\right)}\left[n\right],q^{\left(j\right)}\left[n\right]\right) \end{array}$$ (27)

With the updated communication scheduling factor $\lambda_{k}^{\left(j+1\right)}\left[n\right]$ and $q^{\left(j\right)}\left[n\right]$, solving the transmission power optimization subproblem yields an updated solution $P_{s}^{\left(j+1\right)}\left[n\right]$ that satisfies


$$\begin{array}{c} R\left(\lambda_{k}^{\left(j+1\right)}\left[n\right],P_{s}^{\left(j\right)}\left[n\right],q^{\left(j\right)}\left[n\right]\right)\leq R\left(\lambda_{k}^{\left(j+1\right)}\left[n\right],P_{s}^{\left(j+1\right)}\left[n\right],q^{\left(j\right)}\left[n\right]\right) \end{array}$$ (28)

Finally, with the updated transmission power $P_{s}^{\left(j+1\right)}\left[n\right]$, and the communication scheduling factor $\lambda_{k}^{\left(j+1\right)}\left[n\right]$, solving the trajectory optimization subproblem gives $q^{\left(j+1\right)}\left[n\right]$ such that


$$\begin{array}{c} R\left(\lambda_{k}^{\left(j+1\right)}\left[n\right],P_{s}^{\left(j+1\right)}\left[n\right],q^{\left(j\right)}\left[n\right]\right)\leq R\left(\lambda_{k}^{\left(j+1\right)}\left[n\right],P_{s}^{\left(j+1\right)}\left[n\right],q^{\left(j+1\right)}\left[n\right]\right) \end{array}$$ (29)

From the above inequalities, the relationships indicate that the value of the objective function *R* is non-decreasing during the iteration process. Under the constraints of flight velocity, acceleration, initial and final positions, and energy, the proposed algorithm is convergent.

**Algorithm Complexity**: The algorithm complexity analysis is as follows. ${𝒫}_{1.1b}$ and ${𝒫}_{1.2b}$ are linear programs. ${𝒫}_{1.1b}$ has *N* + 1 variables and *N* + 2 linear constraints, with a computational complexity of *O*(*N*^3.5^). ${𝒫}_{1.2b}$ has the same number of variables but only two linear constraints, yielding the same asymptotic complexity of *O*(*N*^3.5^) with a much smaller constant factor. ${𝒫}_{1.3c}$ is a second-order cone program with (8 + *M* + *K*)*N* + 1 variables, *M* + *K* + 2 second-order cone constraints of size *N* + 1, and 3*N* + 5 linear inequalities. Since *M* + *K* is small (constant with respect to N), its complexity reduces to *O*(*N*^3.5^) as well. Overall, all three problems share the same asymptotic complexity of $O(I_{Iter}N^{3.5}\log\left(\frac{1}{\varepsilon }\right))$, $I_{Iter}$ is the number of iteration.

## 4 Numerical results

The relevant parameters were given out and the simulation result is to illustrate the effectiveness of our proposed algorithm. This system consists of *K* = 4 GNs and *M* = 3 lasers. The other simulation parameters are given as Table 3.

**Table 3:** Simulation parameters.

Parameters	Value	Parameters	Value
$q_{k}$	[50,300], [300,350], [450,200], [200,50]	$q_{m}$	[100,200], [250,200], [400,200]
$q_{I}/q_{F}$	[0,100] / [500,300]	H	100 m
$\beta_{0}$	−50 dB	$\sigma^{2}$	−80 dBm
*P*_0_	74.68 W	$P_{i}$	118.21 W
$U_{tip}$	120 m/s	*v*_0_	5.38 m/s
*d*_0_	0.50	ρ	1.225 kg/m^3^
*s*	0.083 m^3^	*A*_0_	0.283 m^2^
*C*	0.004	φ	800 W
*D*	0.1	Δθ	$3.4*10^{-5}$
α	10^−5^	$v_{\max}$	40 m/s
$a_{\max}$	5 m/s^2^	*P*	30 dBm

To illustrate our algorithm, we design two comparative benchmarks: Benchmark 1: Adopts fixed power allocation with time slots evenly distributed in node sequence, focusing exclusively on trajectory optimization. Benchmark 2: Employs a fixed UAV trajectory, with optimization limited to GNs communication scheduling and power allocation.

**Fig 2:**
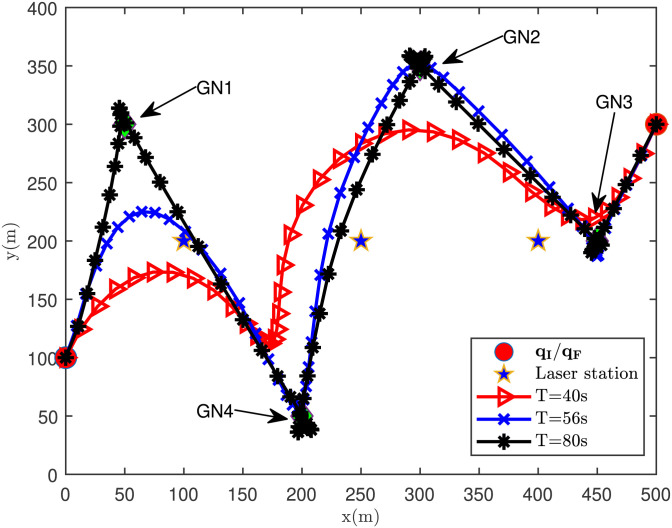
Flying trajectory versus different T.

Fig 2 plots the trajectory versus different *T*. The UAV traverses from $q_{I}$ to $q_{F}$ within the given time *T*. In order to improve the throughput, the UAV flies as close as possible to GNs for communication, and to receive as much laser power supply as possible, the UAV passes as close as possible to the laser transmitting station. The more time, the UAV flying closer to the first GN. It can be seen from the figure that as the given time increases, the UAV flies closer to the GN and hover above the GNs to increase the throughput. The trajectory while flying from the first GN to the other GNs is the same except for the hovering time in the vicinity, which is due to the fact that the closer to the GN, the longer the communication time and the higher the throughput.

**Fig 3:**
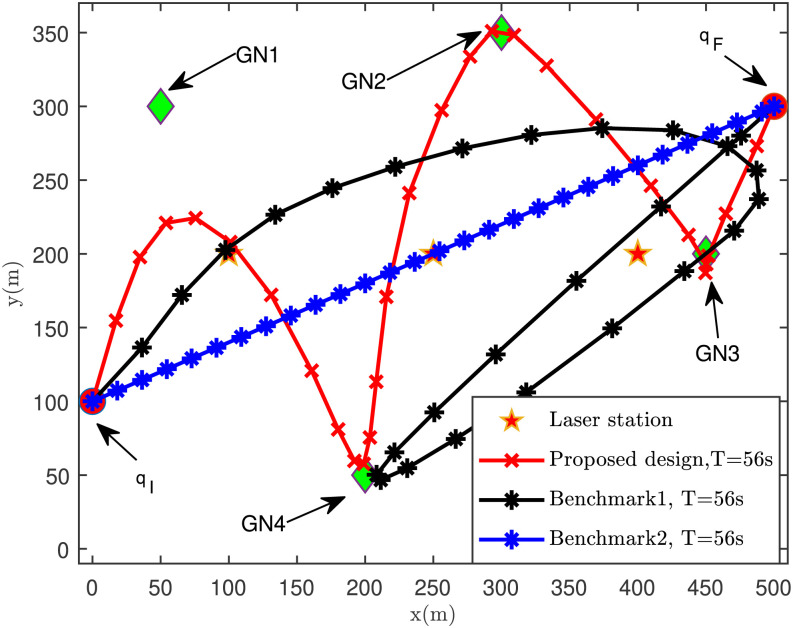
Flying trajectory versus different schemes.

Fig 3 plots the trajectory versus different schemes. As shown in Fig 3, the trajectory characteristics of the three schemes are as follows: Benchmark 1 traverses sequentially in node order and finally returns to the $q_{F}$; Benchmark 2 employs a straight-line trajectory from the $q_{I}$ to the $q_{F}$; our proposed algorithm approaches each node as closely as possible during flight to enhance information transmission. Among them, Benchmark 1 can only perform traversal according to the preset node order due to the lack of a GN scheduling mechanism.

**Fig 4:**
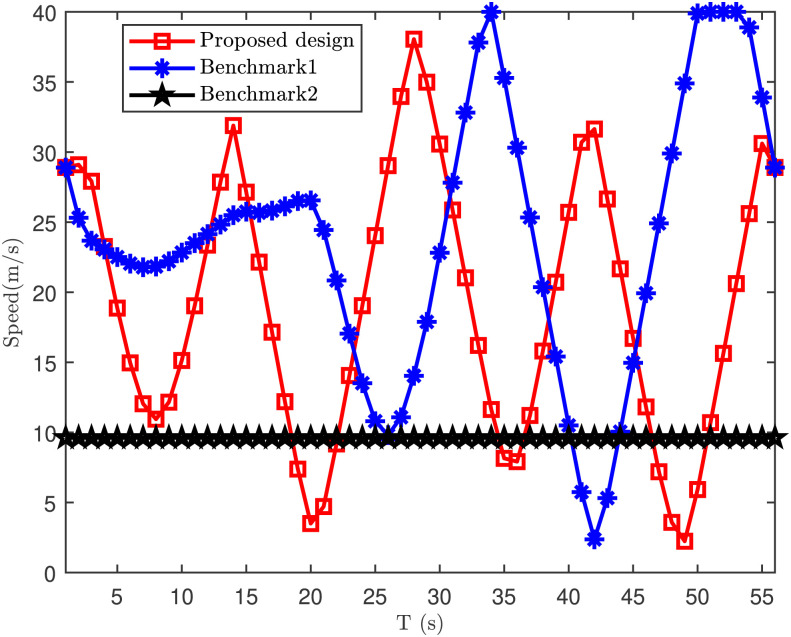
UAV speed for *T* = 56 s.

**Fig 5:**
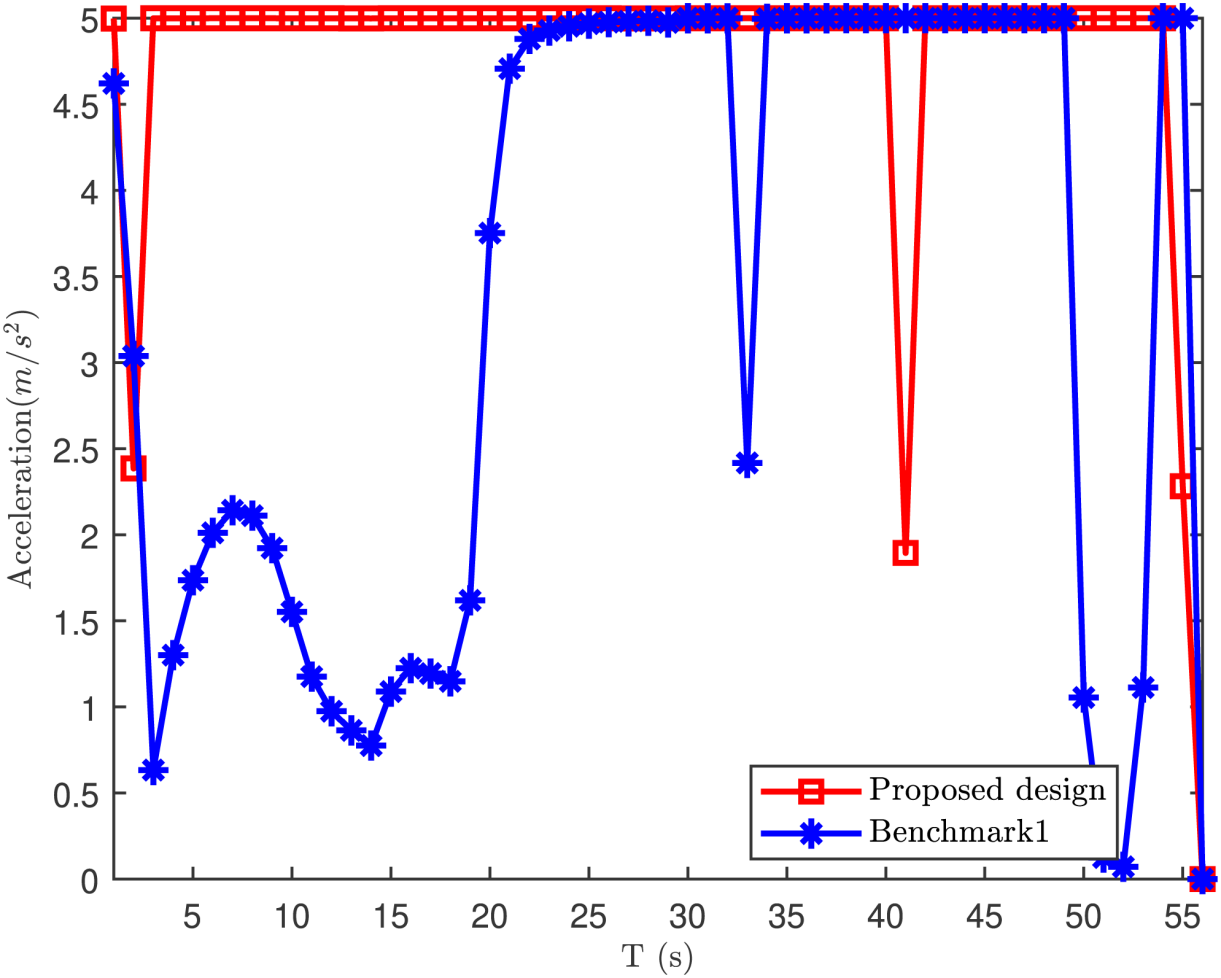
UAV accelerate for *T* = 56 s.

Figs 4 and 5 depict the speed and acceleration of the UAV at *T* = 56 s. It can be seen that proposed algorithm at the beginning the UAV flies the GN fast and then the speed decreases to increase the hovering time which communicates with the GNs in close proximity in Fig 4. Our proposed algorithm exhibits higher speed and acceleration than Benchmark 1 during most time intervals. Furthermore, Benchmark 2 maintains a constant speed throughout its flight to the destination. Fig 5 shows the UAV’s acceleration profile: rapid acceleration towards GNs followed by deceleration to prolong hovering time. The UAV has higher speed and acceleration when flying between GNs, and lower speed and acceleration when hovering near the GN.

**Fig 6:**
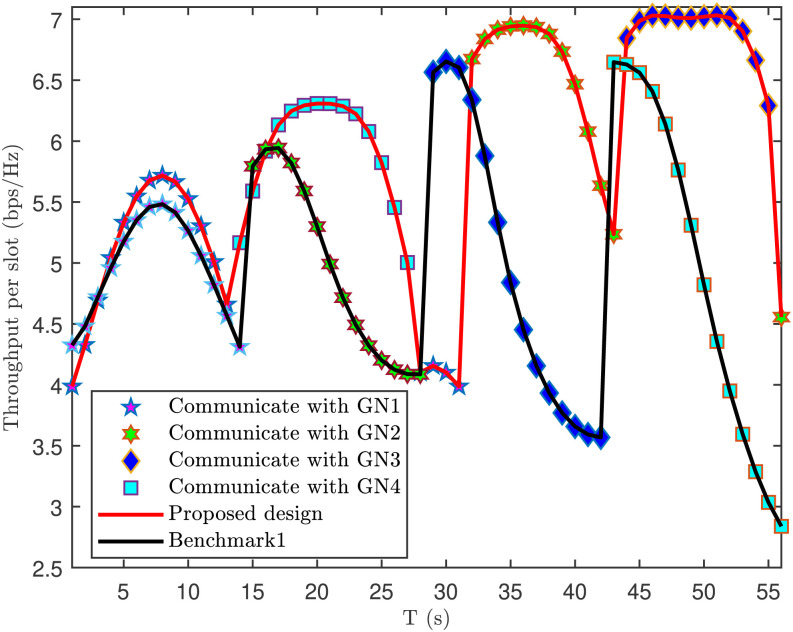
Throughput per slot for *T* = 56 s.

Fig 6 shows the throughput of the UAV per time slot, the different colors are utilized to mark the communication schedule. It is clear from the figure that the throughput remains essentially constant in the vicinity of GNs. The communication scheduling between the UAV and the GNs is not exactly sequential, after communicating with the first GN, it continues to communicate with the fourth GN, while after communicating with the fourth GN, it continues to communicate with the first GNs. As shown in Fig 6, our proposed algorithm achieves a higher rate than Benchmark 1 within the time slot. This is attributed to the fact that GN scheduling and power optimization can effectively enhance the fairness among the GNs.

**Fig 7:**
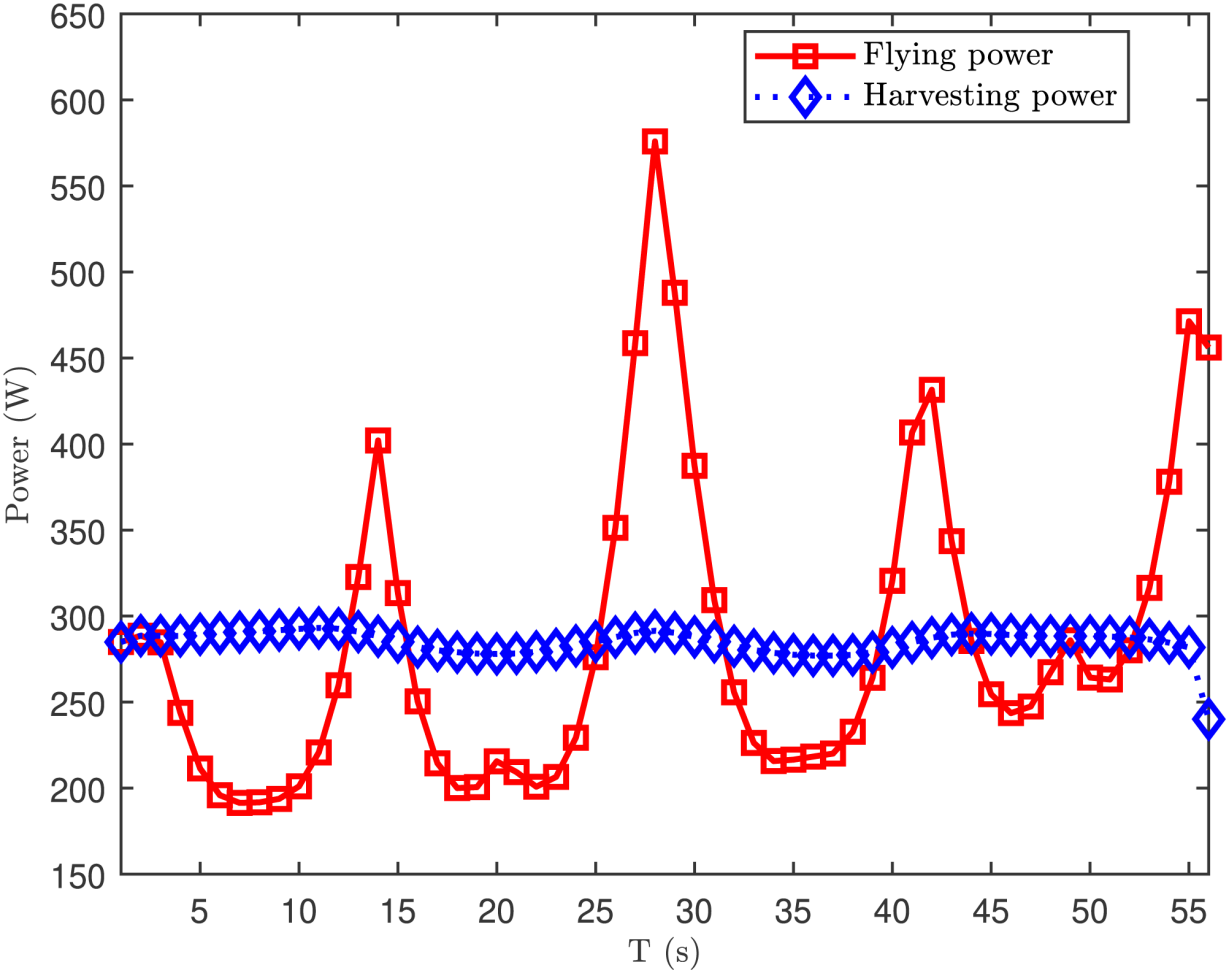
Power for flying and harvesting in each slot.

Fig 7 shows the UAV flight power and the harvested power of our proposed design with *T* = 56 s in each slot. As can be seen from the figure, the flight power is lower than the harvested energy most of the time during the flight, and it is only when flying towards the GNs that the flight is carried out at a high power, which corresponds to the Fig 4 of the UAV flight speed.

**Fig 8:**
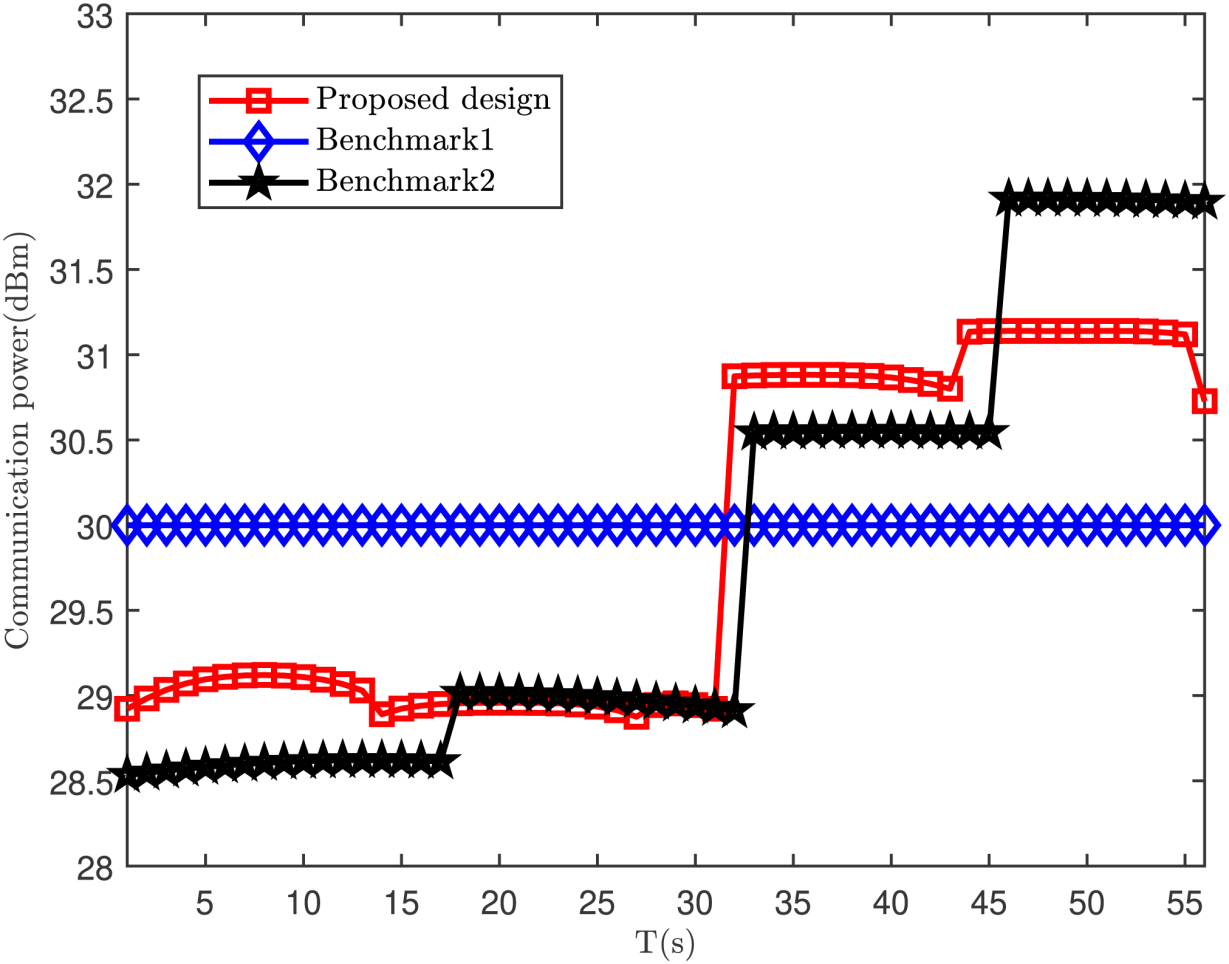
Communication power.

Fig 8 shows the communication transmission power of the UAV with *T* = 56 s. As shown in the figure, our proposed algorithm and Benchmark 1 exhibit lower transmission power during most time intervals. This is because the total communication energy is limited, and transmitting at a higher power during part of the time slot can effectively improve the GN throughput rate when close the GN.

**Fig 9:**
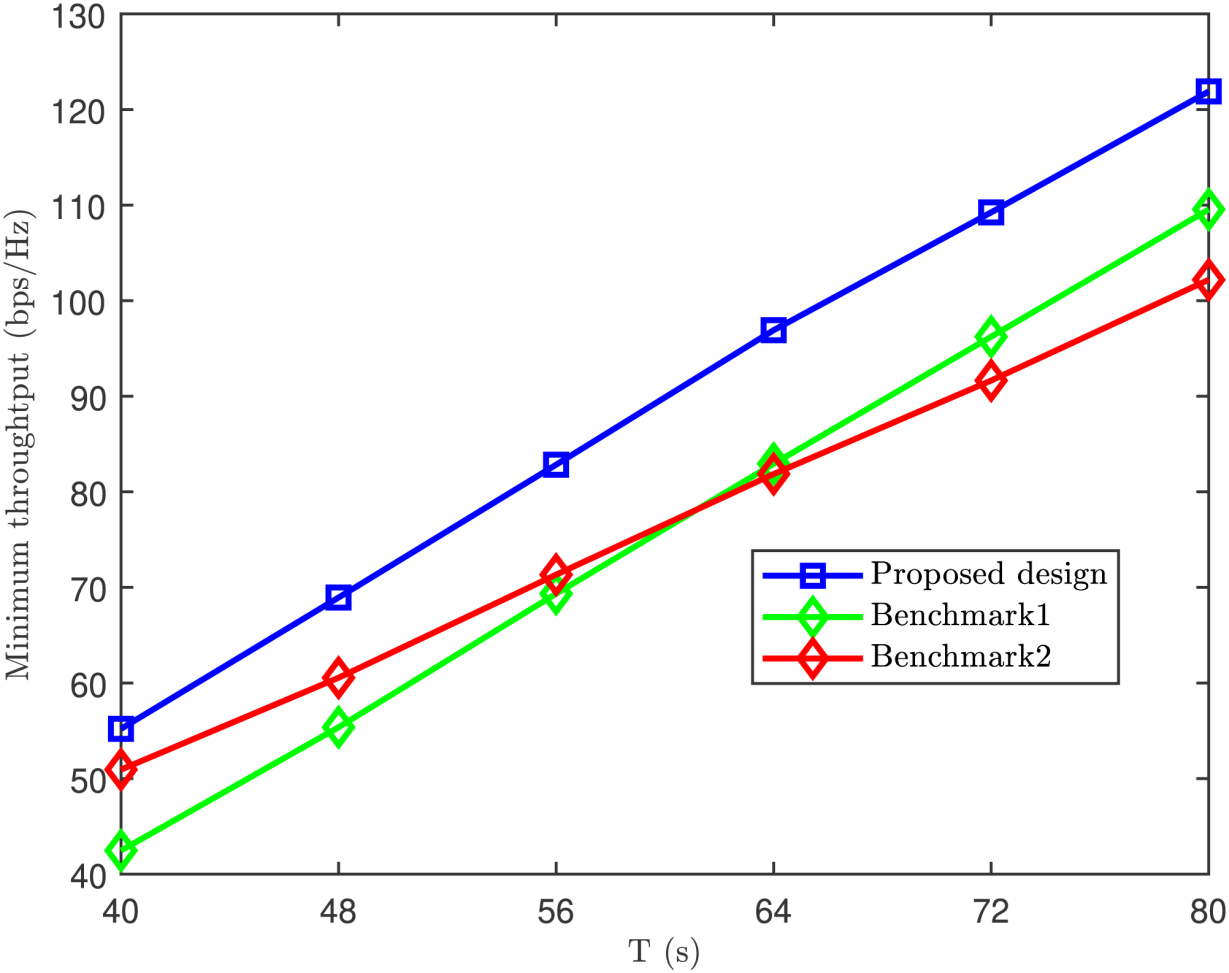
Minimum throughput versus varying *T.*

Fig 9 presents the minimum throughput of the three different schemes versus different *T*. It can be seen that the minimum throughput of the three scenarios increases with the increase of the given time. Our proposed algorithm increases significantly higher than the other two schemes the longer the given time.

**Fig 10:**
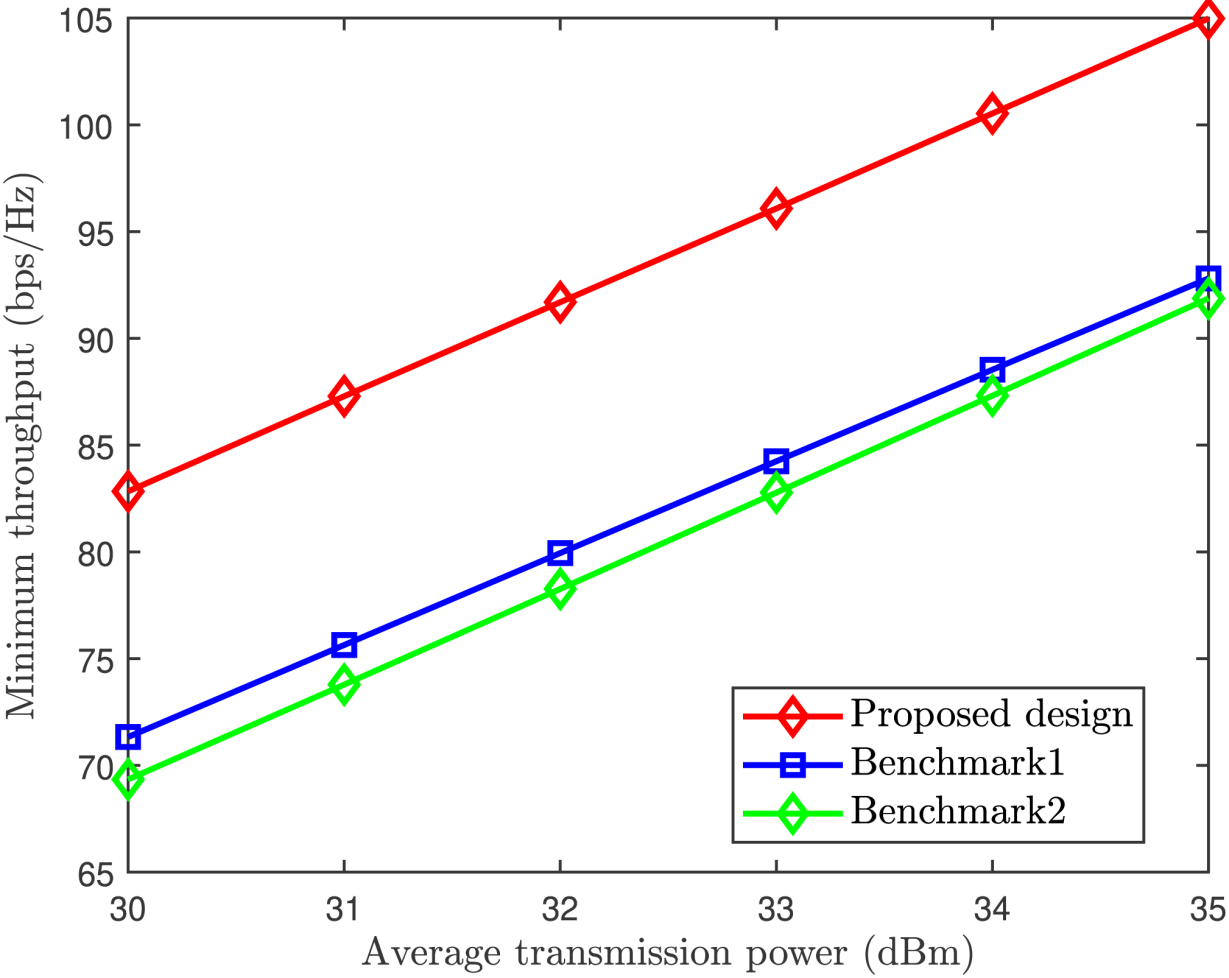
Minimum throughput versus varying *P.*

Fig 10 shows the minimum throughput of the three different scenarios versus different *P*. The minimum throughput of the three scenarios increases with the increase of the given power. Our proposed algorithm increases significantly higher than the others.

**Fig 11:**
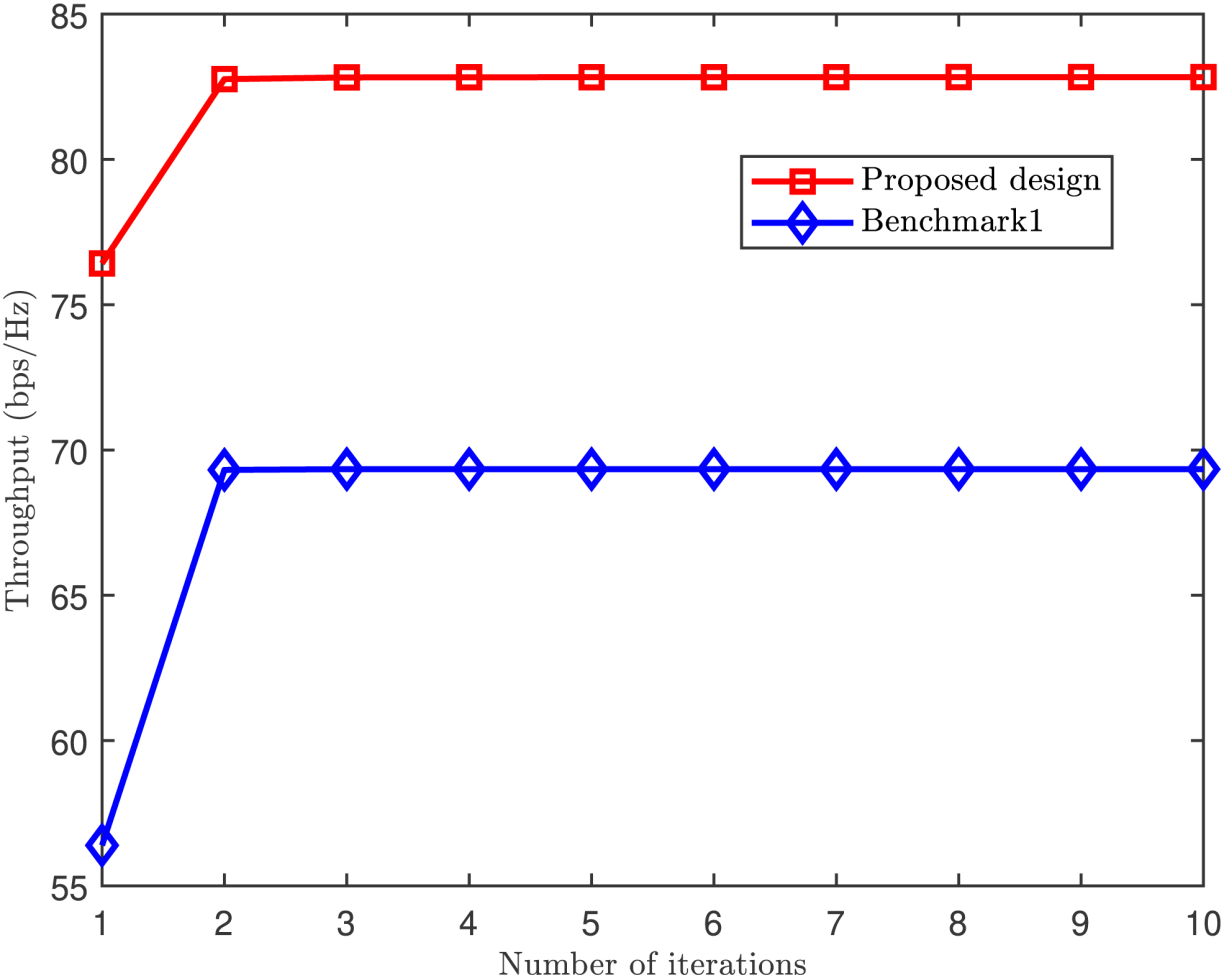
Converge rate of the proposed algorithm.

Fig 11 plots the convergence of the proposed algorithm with *T* = 56 s. We can see from the figure that the algorithm converges gradually after four iterations and the smoothness after convergence is better, which indicates that our proposed algorithm has a faster convergence speed and better convergence.

## 5 Conclusion

In this paper, we investigate max-min fair throughput optimization in laser-powered UAV systems. The original problem is rather difficult to solve. We adopt the BCD technology to transform the original problem into three sub-problems: communication scheduling, transmission power optimization, and flight trajectory optimization. The iterative method is adopted to finally obtain the optimized solution. Issues such as the battery capacity of laser-powered UAV and dynamic switching among multiple laser stations will be left to our future work.
